# Supplementary material for: Heparin-based hydrogel scaffolding alters the transcriptomic profile and increases the chemoresistance of MDA-MB-231 triple-negative breast cancer cells
Source: Biomater Sci. 2020 Feb 13;8(10):2786–96. doi: 10.1039/c9bm01481k (PMC7497406; doi:10.1039/c9bm01481k)
Supplement: Supplementary file 2 [file BM-008-C9BM01481K-s002.zip › Supplementary File 4/HP-BhydrogelvControl/Pathways/my_analysis.Gsea.1545201079907/HALLMARK_HEDGEHOG_SIGNALING.html]

Details for gene set HALLMARK\_HEDGEHOG\_SIGNALING[GSEA]

|  || Dataset | expr.class.cls#HYD\_versus\_CONTROL.class.cls#HYD\_versus\_CONTROL\_repos |
| Phenotype | class.cls#HYD\_versus\_CONTROL\_repos |
| Upregulated in class | HYD |
| GeneSet | HALLMARK\_HEDGEHOG\_SIGNALING |
| Enrichment Score (ES) | 0.2026864 |
| Normalized Enrichment Score (NES) | 0.5896352 |
| Nominal p-value | 0.9681335 |
| FDR q-value | 0.99555016 |
| FWER p-Value | 1.0 |
Table: GSEA Results Summary

  

Fig 1: Enrichment plot: HALLMARK\_HEDGEHOG\_SIGNALING      
 Profile of the Running ES Score & Positions of GeneSet Members on the Rank Ordered List

  

| PROBE | DESCRIPTION (from dataset) | GENE SYMBOL | GENE\_TITLE | RANK IN GENE LIST | RANK METRIC SCORE | RUNNING ES | CORE ENRICHMENT || 1 | CELSR1 | na |  |  | 625 | 2.178 | 0.0941 | Yes |
| 2 | CRMP1 | na |  |  | 2263 | 1.253 | 0.0816 | Yes |
| 3 | LDB1 | na |  |  | 2963 | 1.046 | 0.1060 | Yes |
| 4 | TLE1 | na |  |  | 3083 | 1.014 | 0.1588 | Yes |
| 5 | TLE3 | na |  |  | 4522 | 0.708 | 0.1249 | Yes |
| 6 | RTN1 | na |  |  | 4735 | 0.670 | 0.1529 | Yes |
| 7 | PTCH1 | na |  |  | 5058 | 0.618 | 0.1720 | Yes |
| 8 | UNC5C | na |  |  | 5729 | 0.522 | 0.1674 | Yes |
| 9 | PML | na |  |  | 5893 | 0.500 | 0.1880 | Yes |
| 10 | ETS2 | na |  |  | 6164 | 0.462 | 0.2008 | Yes |
| 11 | NRP2 | na |  |  | 6663 | 0.397 | 0.1980 | Yes |
| 12 | NF1 | na |  |  | 6979 | 0.364 | 0.2027 | Yes |
| 13 | CDK5R1 | na |  |  | 7644 | 0.286 | 0.1847 | No |
| 14 | NRCAM | na |  |  | 9653 | 0.080 | 0.0846 | No |
| 15 | CDK6 | na |  |  | 10768 | -0.034 | 0.0285 | No |
| 16 | SHH | na |  |  | 10965 | -0.061 | 0.0218 | No |
| 17 | L1CAM | na |  |  | 11933 | -0.167 | -0.0189 | No |
| 18 | NRP1 | na |  |  | 13560 | -0.362 | -0.0827 | No |
| 19 | ACHE | na |  |  | 13587 | -0.367 | -0.0627 | No |
| 20 | RASA1 | na |  |  | 14048 | -0.423 | -0.0620 | No |
| 21 | PLG | na |  |  | 14094 | -0.429 | -0.0394 | No |
| 22 | DPYSL2 | na |  |  | 15803 | -0.704 | -0.0876 | No |
| 23 | OPHN1 | na |  |  | 17263 | -1.072 | -0.1013 | No |
| 24 | NKX6-1 | na |  |  | 17360 | -1.105 | -0.0420 | No |
| 25 | VLDLR | na |  |  | 17499 | -1.153 | 0.0178 | No |
| 26 | SCG2 | na |  |  | 17637 | -1.216 | 0.0814 | No |
Table: GSEA details [plain text format]

  

Fig 2: HALLMARK\_HEDGEHOG\_SIGNALING      
 Blue-Pink O' Gram in the Space of the Analyzed GeneSet

  

Fig 3: HALLMARK\_HEDGEHOG\_SIGNALING: Random ES distribution      
 Gene set null distribution of ES for **HALLMARK\_HEDGEHOG\_SIGNALING**
